# A call for the standardised reporting of factors affecting the exogenous loading of extracellular vesicles with therapeutic cargos

**DOI:** 10.1016/j.addr.2021.04.012

**Published:** 2021-06

**Authors:** Stephanie Rankin-Turner, Pieter Vader, Lorraine O'Driscoll, Bernd Giebel, Liam M. Heaney, Owen G. Davies

**Affiliations:** a School of Sport, Exercise and Health Sciences, Loughborough University, Loughborough, Leicestershire LE11 3TU, UK; b CDL Research, University Medical Centre Utrecht, Heidelberglaan 100, 3584 CX Utrecht, The Netherlands; c Department of Experimental Cardiology, University Medical Centre Utrecht, Heidelberglaan 100, 3584 CX Utrecht, The Netherlands; d School of Pharmacy and Pharmaceutical Sciences & Trinity Biomedical Sciences Institute, Trinity College Dublin, Dublin, Ireland; e Trinity St. James’s Cancer Institute, Trinity College Dublin, Dublin, Ireland; f Institute for Transfusion Medicine, University Hospital Essen, University of Duisburg-Essen, Virchowstraβe 179, 45147 Essen, Germany

**Keywords:** Extracellular vesicles, Exosomes, Microvesicles, Loading, Drug delivery, Guidelines, Efficiency

## Abstract

Extracellular vesicles (EVs) are complex nanoparticles required for the intercellular transfer of diverse biological cargoes. Unlike synthetic nanoparticles, EVs may provide a natural platform for the enhanced targeting and functional transfer of therapeutics across complex and often impenetrable biological boundaries (e.g. the blood–brain barrier or the matrix of densely organised tumours). Consequently, there is considerable interest in utilising EVs as advanced drug delivery systems for the treatment of a range of challenging pathologies. Within the past decade, efforts have focused on providing standard minimal requirements for conducting basic EV research. However, no standard reporting framework has been established governing the therapeutic loading of EVs for drug delivery applications. The purpose of this review is to critically evaluate progress in the field, providing an initial set of guidelines that can be applied as a benchmark to enhance reproducibility and increase the likelihood of translational outcomes.

## Graphical abstract

**Figure:**
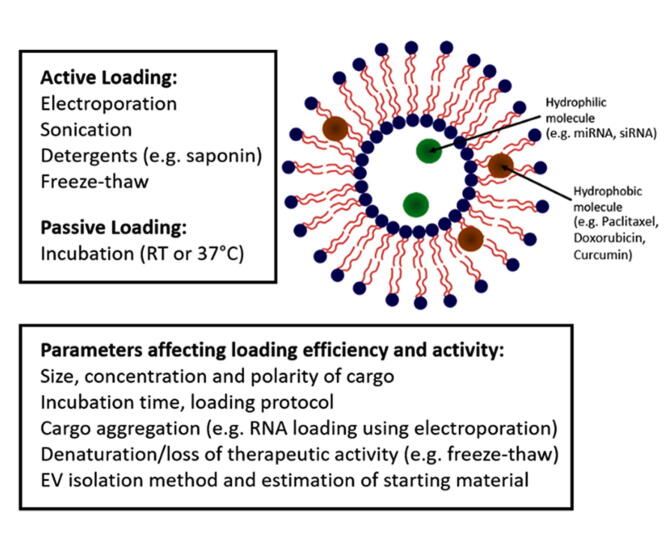

## 1 Introduction

Extracellular vesicles (EVs) are lipid-enclosed biological nanoparticles that are released by practically all living cells. Initially believed to simply be a route of exporting cellular waste, the importance of EVs in a variety of physiological and pathological intercellular signalling events is now widely appreciated.[1], [2] Unsurprisingly, the capacity of EVs to provide a biologically analogous environment for the selective transfer of a diverse array of biological molecules to specific tissues and cell types has raised considerable interest in their application as advanced drug delivery systems (DDS). To date, the number of studies seeking to load EVs with exogenous therapeutic cargoes is expanding, with an almost 5-fold increase in publications observed since 2015 (Source: PubMed search terms: “extracellular vesicles” or “exosomes” and “loading”, Dec 2020). This increase has been most evident for the encapsulation of small RNAs [3], [4], [5], [6] and chemotherapeutic drugs such as doxorubicin [7], [8], [9]. Such advancements have considerable implications since they could provide a means of improving the penetration of approved chemotherapeutics within the densely packed extracellular matrix of solid tumours, where a recent *meta*-analysis has highlighted that only 0.7% penetration is achieved using conventional synthetic nanoparticle systems.[10] The emergence of EV DDS also has considerable implications for the delivery of high molecular weight biological drugs such as proteins, peptides or RNA therapies, which now account for over 93% of net drug spending at a global level.[11] These therapies are currently administered parenterally, with short plasma half-lives and poor patient compliance often resulting from the required frequency of administration. Furthermore, in the absence of an effective delivery system, the administration of biologics can elicit a host inflammatory response and provides no means of ensuring the drug reaches its intended intracellular target in an active state and at the required dose.[12] This is particularly true for RNA therapeutics, where efficacy is reliant on the biological drug (e.g. mRNA) reaching its intended cellular target and influencing translational events in order to deliver a therapeutic effect. Consequently, advancements in DDS that take advantage of the naturally endowed properties of EVs could provide an emerging drug delivery system reduces the risk of immunological or toxic side effects, while providing an evolutionarily advanced mechanism for the secure and targeted delivery.

It is evident that EVs hold considerable promise as a next generation DDS. However, despite continued growth in the number of EV publications, we must remain aware that the field is only just beginning to be established. Although EV-like particles were originally identified in 1946, the first indication that these particles could mediate functional effects was not described until 1996, when Raposo and colleagues documented that MHC class II containing intraluminal vesicles from B lymphocytes could regulate the activity of T cells.[13] This discovery was followed by the studies in 2006 and 2007, in which functional horizontal RNA transfer was observed between EVs and recipient cells.[14], [15] Several years later, these initial discoveries were furthered by a publication from Matthew Wood’s group that reported intravenously administered siRNA loaded EVs could be targeted specifically to neurons, microglia and oligodendrocytes in the brain, resulting in the downregulation of the therapeutic Alzheimer’s target gene BACE1.[16] At the time EVs were referred to non-specifically as exosomes, microvesicles etc, with limited understanding of the differential effects of these EV subsets. With the aim of promoting standardisation in the field, the International Society for Extracellular Vesicles (ISEV) was established in 2011 and the first position statement on the minimal information for studies of extracellular vesicles (MISEV) was published in 2014.[17] In this publication, it was suggested that due to a lack of specific markers able to accurately distinguish distinct vesicle subsets, that all cell-derived particles be commonly referred to as EVs to ensure standard reporting in the field. The publication also introduced an initial recommended criteria for the general characterisation of EVs and was further expanded in 2018 as the field advanced.[18]

With interest in the field continuing to grow, ISEV launched an online survey in 2017 to assess the primary needs and concerns of the rapidly expanding EV research community.[19] Amongst several points of concern raised by respondents, EV cargo loading and transfer were consistently identified as significant obstacles in the field - with a specific focus on RNA loading. In the comprehensive 2018 MISEV guidelines, ISEV published expanded guidelines for authors that detailed numerous pertinent aspects of EV science and provided a non-exhaustive list of methods and protocols that could be applied for their isolation/separation. However, despite previously acknowledging widespread limitations relating to EV loading, there is a lack of guidelines governing EV loading studies. Furthermore, a lack of standardisation in the methodology and reporting of EV loading parameters can be widely observed throughout the literature, with considerable variation evident in several fundamental parameters (Table 1). These include:

• Variations in EV starting material – typically assessed by total protein concentration, sample volume or particle number.
• Protocols applied for the loading of cargo –these can be passive or active.
• Estimation and reporting of variables such as EV loading capacity and efficiency.

**Table 1:** Summary of studies employing different EV loading strategies.

**Cargo**	**Loading Method**	**Species**	**Loading Efficiency/Capacity**	**References**
Small RNA and Oligonucleotides (miRNA & siRNA)	Electroporation	Bovine, Murine, E. coli, Human	<0.5–62%	[3], [4], [5], [6], [16], [68], [75], [76], [78], [79], [84], [85], [90], [91], [92], [96], [99], [114], [115], [116], [117], [118], [119], [120], [121], [122], [123], [124], [125], [126], [127]
	Chemical Transfection	Bovine, Human, Murine	NR-30%	[6], [76], [127]
	Sonication	Human	NR	[68]
	Incubation	Human, Murine	70–80%	[58], [59], [60], [128]
Small Molecule Drugs	Electroporation	Human, Murine	0.5–50%	[7], [8], [9], [64], [67], [80], [116], [129]
	Sonication	Human, Murine	8–30%	[67], [93], [130], [131], [132]
	Incubation	Bovine, Human, Murine	1–67%	[28], [50], [51], [61], [64], [67], [129], [131], [132], [133]
	Thermal Shock	Human	10–13%	[61], [132]
DNA and Plasmids	Electroporation	Human, Murine	1.75–20%	[83], [134], [135]
Proteins (e.g. Enzymes and Inhibitors)	Electroporation	Bovine, Human	0.4–0.5%	[81], [82], [136], [137], [138]
	Incubation	Murine	5–19%	[54], [139]
	Sonication	Murine	26%	[54], [139]
	Thermal Shock	Murine	15%	[54]
	Extrusion	Murine	22%	[54]
Nanoparticles	Electroporation	Murine	7%	[63]
	Sonication	Murine	19%	[63]
	Incubation	Murine	14–16%	[63]
	Thermal Shock	Murine	9–18%	[63]
	Chemical Transfection	Human	NR	[140]
Gene-Editing Complexes	Electroporation	Human	NR	[141]

This review will focus on the growing number of studies that have utilised passive or active methods for the exogenous loading of EVs (see Table S1 for a comprehensive overview), it is also important to acknowledge that endogenous loading methods are being developed in an attempt to improve efficacy and reproducibility. These methods seek to exploit a cells endogenous machinery for the loading of biological cargos manufactured inside the nucleus, which are subsequently loaded into multivesicular bodies (MVBs) or at the plasma membrane for release into the extracellular environment via EVs.[20] While we now have a basic understanding of some of the underlying intracellular processes governing the sorting and integration of biological material in endosomal vesicles (e.g. RNA-binding proteins such as Y-box protein 1 or Exportin-5), it should be acknowledged that the precise mechanisms governing EV cargo loading remains poorly defined.[21], [22] Furthermore, endogenous loading is only applicable for the encapsulation of biological molecules and cannot realistically be applied for the reproducible incorporation of chemically synthesised drugs. Consequently, there exists a clear need to define reproducible protocols for the exogenous loading of EVs. While we acknowledge that variation in some experimental parameters may in part be due to a lack of specialist knowledge or equipment, considerable variation can nonetheless be observed across studies applying the same isolation technique (Table 1 and Table S1). As such, numerous unanswered questions exist around parameters required for the effective exogenous loading of EVs. While protocols will need to be tailored to account for inherent differences in molecular weight, polarity and the wider biochemical properties of the cargo in question,[23] it remains too early to tell whether these protocols will also need to account for potential compositional differences related to EV biogenesis (e.g. exosomes and microvesicles), cellular origin and/or microenvironment. Undoubtedly, these variations will have an effect on the precise lipid and protein composition of EVs, as well as variations in native cargo loads and the downstream effect this could have on loading capacity. As such, precise questions relating to optimal requirements for the loading of EVs and whether these protocols will likely need to be bespoke for each drug/biomolecule/EV remain largely unanswerable at this early stage.

In this review, we highlight a pressing need for the standardised reporting of data in this burgeoning and potentially impactful area of therapeutic science. The review details current methodologies applied for the loading of EVs (Table 1) and identifies an urgent need for the implementation of standardised reporting in the field. To assist with this endeavour, we provide the first basic framework outlining recommended reporting criteria for studies involving EV loading, which has been designed to supplement existing ISEV guidelines and position statements to enhance best practice in therapeutic EV science that help advance the field toward clinical application.[24], [25] Publications included in this review were primarily identified using PubMed with the search terms “extracellular vesicles” or “exosomes” and “loading”. Throughout the review, loading efficiency relates to the percentage of total available drug that has been encapsulated within EVs, whereas loading capacity refers to the amount of drug loaded per mass of particles.

## 2 EV isolation and quantification

**Fig. 1:**
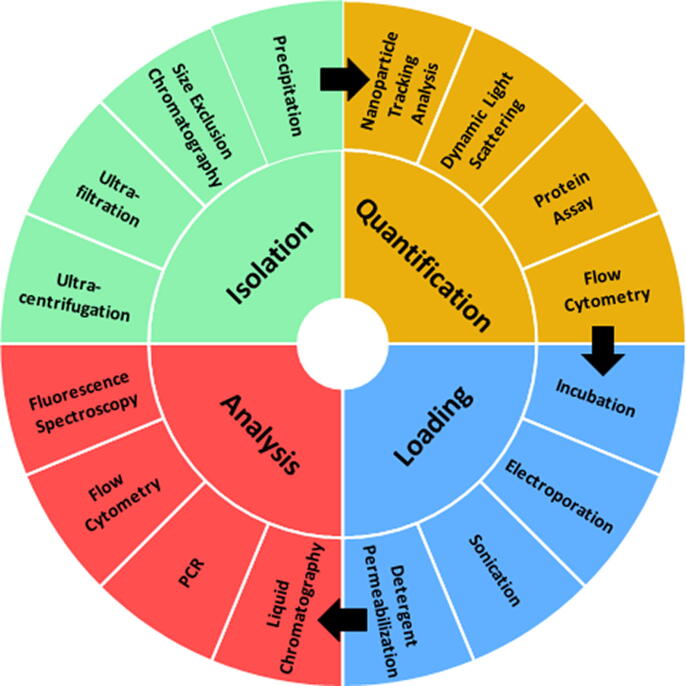
Summary of the variety of techniques utilised in loading studies for the isolation, quantification, loading and analysis of EVs.

EVs are commonly derived from cell culture media and biological fluids such as urine,[26], [27] milk,[6], [28] and plasma.[4], [29], [30] Purification is necessary to remove the majority of cell debris and other non-EV contaminants, with various methods available to achieve this aim (Fig. 1). Since the origin of EVs is likely to dictate their composition, physicochemical properties and tissue/cell selectivity, EV isolation for drug delivery is likely to depend on the precise therapeutic target in question. Consequently, within the literature there are examples of EVs being isolated from a diverse range of cell types, biofluids and cell culture media (Table 1 and Table S1). Common isolation methods include differential ultracentrifugation (UC), size-exclusion chromatography (SEC), ultrafiltration (UF) and polymer-mediated precipitation, while alternative approaches such as tangential flow filtration (TFF), density gradient centrifugation and immunoaffinity isolation can help provide increased purity or specificity (Fig. 1).[31], [32] For a comprehensive overview of each, the authors recommend the following review on the technical challenges of working with EVs.[33]

In the context of EV therapeutics culture conditions, medium harvesting intervals and EV preparation technologies have considerable influences on product identities including EV composition, purity and non-EV associated by-products.[34] Consequently, upstream and downstream processing methods will inevitably influence the efficiency and reproducibility of any downstream EV loading protocol. Furthermore, applied quality control procedures can affect conclusions about the composition of end-products. For example, assessing EV recovery using a protein quantification assay is likely to provide limited information on total EV concentration unless co-precipitated proteins/lipoproteins can be efficiently removed (e.g. using a density gradient or filtration step), while particle counts as determined by scattering techniques do not necessarily provide an accurate measure of EV concentration (as small EVs remain largely undetected) and can be operator-dependent.[35] To account for fundamental differences in starting EV concentration, it is essential that standard reporting guidelines are implemented to ensure basic comparisons between studies employing a diverse range of isolation protocols can be made.[36] For example, this could be through calculation of the particle to protein ratio to provide an indication of sample purity or via the quantification of well described EV markers such as the tetraspanins CD9, CD63 or CD81 (e.g. by flow cytometry). However, the authors acknowledge the variability of such measures when analysing EVs obtained from different cell types and biofluids. We acknowledge that is information is likely to be variable amongst studies and recommend that at least a basic level of sample characterisation is conducted. As the field progresses, it is likely that reference materials (e.g. recombinant EVs) with comparable sizes, epitopes and refractive indeces will become available for the calibration, validation and quality control of EV measurements.[37] It is also important that the benefits and limitations of the isolation method applied is well described to provide a transparent overview of the study and its findings. We shall now consider some of the limitations of routinely applied EV isolation methods such as UC and SEC - and how these limitations could impact exogenous loading - in greater detail.

UC is a cost-effective and widely utilised method for the isolation of EVs. However, this method has a relatively low recovery rate and pellets the EV fraction through the application of high g-forces (≥100,000 xg) that have previously been shown to introduce a degree of disruption and EV aggregation.[38], [39] When applying UC for the isolation of EVs, authors should acknowledge the potential for operator-dependent variations in EV recovery, with the type of centrifuge and rotor found to have an impact.[40], [41] The process of UC is also likely to influence EV membrane integrity, which could subsequently have knock-on effects on factors such as therapeutic loading, retention and reproducibility in drug delivery studies. Of course, it is possible to maintain EV integrity through the application of gentler isolation methods such as SEC, which is often used in combination with UF or tangential flow filtration (TFF) as a means of increasing particle yield, with recovery rates of up to 40% reported.[42], [43] Several studies have demonstrated that the collection of vesicles by SEC can be valuable for retaining increased levels of biological functionality.[42], [44] However, although SEC is being increasingly applied for the isolation of EVs, it is more specialised than UC and requires the purchase or construction of chromatography columns. Furthermore, much like UC, SEC is known to result in the co-isolation of lipoproteins, which can skew downstream measures of protein and particle concentration.[45] SEC is frequently combined with UF in order to concentrate the resulting EV enriched fractions. However, post-SEC concentration using UF can result in a loss of EVs due to their non-specific binding with the filter membrane.[46] As such, it is important that measurements of particle concentration are recorded post-filtration using techniques such as flow cytometry or NTA, and that relative measures of lipoprotein contamination are made available in any resulting publications. If access to such facilities is limited, it is important that proteins indicative of lipoprotein contamination (APO proteins) be assessed using western blotting. We also suggest that filters applied for EV concentration (e.g. Amicon, Vivaspin) are carefully selected and validated before application, since differences in membrane composition could have an impact on vesicle recovery and activity.[46] Lastly, it is possible to increase the specificity and/or reproducibility of EV isolation using approaches such as sucrose density gradient centrifugation and immunoaffinity isolation (e.g. using antibody-coated magnetic beads).[47] However, increased specificity comes at the cost of throughput, potentially limiting scale up in a translational setting. As such, we recognise that such approaches will not always be practical.

As it is not feasible to stipulate that EVs must be enriched using only a specific and sometimes specialised method, when reporting EV loading it is appropriate that minimal information be included to ensure broader reproducibility (Table 2). We recommend that all authors publishing in the field implement the most recent MISEV guidelines, providing full and transparent disclosure on the methods applied for particle isolation and quantification. Where appropriate, authors should centrally record the experimental parameters of their research (e.g. using open access databases such as EV-TRACK, https://evtrack.org/index.php), in order to enhance transparency, critical interpretation and reproduction.[48] Where achievable, researchers should endeavour to provide as quantitative and specific a measure of the number of EVs being loaded as possible, which can only be achieved by incorporating particle counting (e.g. flow cytometry, nanoparticle tracking analysis), immunological (e.g. Western blot, ELISA) and microscopy (e.g. transmission electron microscopy and atomic force microscopy) approaches. Fundamentally, it is essential that authors clearly outline pertinent experimental limitations pertaining to the isolation of EV fractions using their chosen system and how this could impact data obtained in their study. In this endeavour, authors should seek to provide a useful and reproducible measure of particle concentration that carefully accounts for potential limitations of the isolation method applied and the presence of non-EV contaminants such as co-isolated proteins (e.g. UC and precipitation) and lipoproteins (e.g. SEC, UC). An overview of recommendations relating to the isolation of EVs in loading studies is summarised in Table 2.

**Table 2:** Recommended reporting guidelines for EV loading studies. Guidelines are intended for use in addition to existing MISEV criteria on EV production, isolation/separation, and characterisation.

**Experimental Parameter**	**Required Information**
**EV Isolation**	Details of the isolation method applied (e.g. ultracentrifugation, ultrafiltration, SEC) with detailed protocols (including centrifuge model and rotor type, speed and time of centrifugation, brand and pore size of filters, number of centrifugation cycles or filtrations, and storage conditions following isolation, where applicable).
	Where possible, authors should demonstrate that the EV isolation strategy has not caused morphological changes, such as through the application of high g-forces during ultracentrifugation.
**EV Characterisation**	For accurate reporting of EV numbers used in loading studies, EV quantification should ideally be estimated by measurement of particle number, for instance by nanoparticle tracking analysis or nano/high resolution flow cytometry. If protein amount is to be used to quantify EVs, authors should make clear the limitations of quantification by protein content. Also, enrichment of marker proteins should be demonstrated when compared with cell lysates to assess purity.
**EV Loading**	EV/sample mixtures should be precisely reported. Information should include: the amount of molecular cargo, the number of particles/EVs, and the loading solvent (PBS, electroporation butter, etc).
	Full details of loading protocols should be provided: Incubation - temperature and time. Sonication - sonic bath/probe model, sonication settings, sonication time and the number of cycles. Detergent permeabilisation - type of detergent, concentration, temperature and incubation time. Electroporation – type/model of electroporator used, full details of electroporation parameters (e.g. voltage, pulse width, pulse number), and the electroporation buffer used.
	The method applied for the subsequent isolation of loaded EVs should be detailed (ultracentrifugation, ultrafiltration, SEC), including centrifuge model and rotor type, speed and time of centrifugation, the number of wash/filtration steps, and the type of filter used (where relevant).
	Where possible, EV quantification should be performed following isolation of loaded EVs to establish EV loss following ultracentrifugation, filtration, etc.
	Authors should demonstrate, where possible, that EV loading has not caused morphological changes to EVs (e.g. via transmission electron microscopy) or altered the biological properties of the particles (e.g. cellular targeting, uptake, biodistribution).
	Authors should state whether loaded EVs were used immediately for further analysis or stored for subsequent analysis. If the latter, storage conditions and time should be specified.
**Loading Assessment**	Full details of analytical technique used for detection of molecular cargo to assess EV loading should be detailed including, where applicable, instrument type and model, detection parameters, any sample treatment required for analysis.
	Clarify whether loading efficiency was determined via measurement of unloaded cargo (e.g. remaining in supernatant following loaded EV isolation) or via measurement of loaded EVs (e.g. following lysis of loaded EVs to release cargo). One should also discriminate luminal loading from simple association through incubation with relevant enzymes (e.g. RNase) or separation using density gradient UC.
	If applicable, authors should state the protocol used to lyse loaded EVs and, if possible, should demonstrate that any EV lysis procedures (e.g. detergent addition) do not interfere with detection of loaded molecular cargo.
	The success of the loading procedure should be reported in the form of loading efficiency or encapsulation efficiency, including specification of the number of sample replicates and the reproducibility of the loading protocol.
**Functional Delivery**	When loaded EVs are to be applied in *in vitro* or *in vivo* studies, appropriate controls should be conducted. This should include EV only, cargo only, and negative controls, all of which should be subjected to the same treatment as the loaded EVs.
	Authors should demonstrate dose-dependent effects of loaded EVs on an *in vitro* or *in vivo* response.

## 3 Exogenous EV loading strategies

Various techniques for the exogenous loading of cargo into EVs have been developed and assessed, ranging from passive procedures (incubation) to active methods, such as sonication or electroporation. In this section we shall discuss some of the methods commonly applied throughout the literature for the loading of EVs and document loading efficiencies achieved across studies (Table 1 and S1, 64 studies included).

Passive loading can be achieved endogenously by incubating the parent cells with a desired molecular cargo, resulting in EV loading prior to exocytosis. Depending on the nature of the material being packaged, loading can also be achieved through genetically engineering the parental cell line. [49] However, this strategy is not appropriate for the loading of synthetic drugs and it is far more prevalent for passive loading to be achieved exogenously in isolated EV preparations. Passive loading is relatively simple and does not require any specialist equipment. EVs are simply incubated with the cargo in solution, allowing for association with the hydrophobic membrane. For example, Yang *et al* incubated EVs (200 µg/mL) with 2 mg/mL chemotherapy drugs paclitaxel and doxorubicin for 2 h at 37 °C, demonstrating loading amounts of 7.3 ng and 132.2 ng respectively.[50] This study highlighted the varying success of incubation depending on the type of the drug to be loaded, with a notably lower amount of paclitaxel compared to doxorubicin successfully loaded into murine endothelial cell-derived EVs, despite both being hydrophobic molecules of a similar size. Saari *et al* demonstrated the possibility of loading paclitaxel into EVs isolated from prostate cancer cells by incubation for just one hour at 22˚C, achieving an average loading efficiency of 9.2%.[51] In a study by Agrawal *et al*, 8% of paclitaxel was loaded into milk-derived EVs after incubation for 15 min at room temperature,[28] while Kim *et al*, achieved a loading efficiency of only 1.4% when incubating paclitaxel with macrophage-derived EVs at room temperature for 1 h. Notably, not all studies clearly outlined their methodology (for example, exact EV numbers or quantity of chemotherapeutic). Therefore, it is not possible to critically compare the respective outcomes of each method. However, incubation times will likely vary depending on the solubility and size of the molecule in question. For instance, encapsulation of hydrophobic small molecule curcumin within EVs isolated from mouse cancer cells was achieved in just 5 min,[52], [53] with 2.9 g curcumin estimated to be bound to every 1 g of EVs. However, despite the relative simplicity of passive incubation, loading efficiencies reported are often low and active loading methods are generally considered superior.[4], [54]

**Fig. 2:**
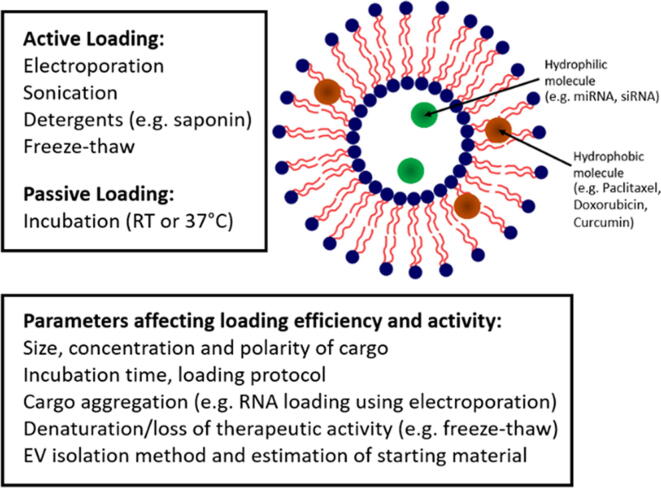
The loading mechanism of therapeutic cargo into extracellular vesicles is dependent on the chemical characteristics of the molecule, with hydrophilic molecules entering the EV lumen and hydrophobic molecules accumulating in the lipid bilayer.

To date, there has been minimal research into the retention of loaded molecules by EVs and it remains unclear how long loaded cargo will remain encapsulated.[55] Consequently, EVs are perhaps unlikely to present an optimal system for the delivery of small molecules that will rapidly dissociate following administration. The propensity for small molecule drugs to passively diffuse out of EVs is also likely to be dependent on their solubility in water, since previous studies have shown that paclitaxel appeared to remain in EVs following loading while the concentration of the more soluble drug doxorubicin was found to have reduced by 70% after 24 h.[51], [56] However, further studies are required to identify precisely which small molecules can be retained and which passively diffuse out following loading. Incubation may also not be suitable for molecular cargos of varying biochemistries without modifying the composition of the loading solution (Fig. 2).[57] Indeed, some studies have proven that modification of the cargo can aid in passive loading. For instance, a hydrophobic moiety can be attached to siRNA to facilitate this process [58], [59], [60]. Through this modification, studies have reported loading efficiencies as high as 80%. However, in this instance the loading efficiency relates to the amount of cargo “associated with” EVs. As such, it includes hydrophobically-modified siRNA that has entered the EV lumen or simply adhered to the membrane surface. This issue was also highlighted by Goh *et al* who, in the loading of U937-derived EVs with doxorubicin, noted that the molecular cargo had a tendency to aggregate and adhere to the vesicle surface, thereby rendering accurate quantification impossible without efficient removal of the bound drug.[61] This type of distinction between EV-loaded and EV-associated cargos could also be applied in many of the previous studies that have been mentioned, and it is important that we are able to distinguish between these two mechanisms if we are really to define effective criteria for the exogenous loading of molecules. For example, the inclusion of an RNase step can be applied for this purpose, thereby degrading any unloaded RNA molecules that have simply associated with the EV rather than luminally incorporated. What is also striking from the studies detailed in Table 1 (also see Table S1 for an additional information) is a lack of investigation into the contribution of basic parameters such as incubation time and temperature, with each study applying varied and largely inconsistent approaches. We propose that in order to devise optimal strategies for the exogenous loading of therapeutic cargoes it is essential that we first advance our understanding of how basic parameters influence uptake and retention for a range of biochemically and diverse molecules.

Although passive loading has been applied in the literature for the loading of small-molecule chemotherapy drugs and curcumin, it is more common for EV loading to be achieved actively via methods, such as electroporation, sonication or chemical permeabilisation using surfactants. In this section we shall provide an overview of active loading methods and give examples of where each has been applied for the encapsulation of exogenous EV cargoes.

Permeabilization of the vesicle membrane can be achieved using detergents such as saponin. This natural surfactant interacts with lipids within the membrane to generate pores for the incorporation of therapeutic cargoes.[62] However, detergent-assisted permeabilisation remains one of the less frequently utilised active strategies for EV loading - applied in only 8% of studies covered in this review. In a study that assessed a range of methods to load the antioxidant enzyme catalase into murine macrophage EVs for use in treatment of Parkinson’s disease,[54] the use of saponin (0.2% solution) was found to improve loading/association (18.5% efficiency) when compared with passive incubation (4.9% efficiency) and freeze/thaw (14.7%) strategies. However, this was reduced when compared with sonication (26.1%). Sancho-Albero *et al* also demonstrated the addition of 0.2% saponin improved the loading of gold nanoparticles into melanoma cell-derived EVs when compared with saponin-free controls, although precise loading efficiencies were not clearly reported in this study.[63] Fuhrmann *et al* were able to demonstrate an 11-fold increase in the loading of hydrophilic porphyrins after just 10 min when incubated in the presence of low concentrations of detergent (as low as 0.01%) when compared with controls lacking surfactant.[64] While a study comparing saponin-assisted permeabilisation and freeze/thaw cycling for the loading of U937-cell derived EVs with doxorubicin reported a maximum efficiency of ~ 50%.[61] Outcomes from these studies are promising, with detergent permeabilization offering a simple and cost-effective means of loading cargo into EVs, without the need for specialist equipment. In addition, the authors of these studies were able to demonstrate that saponin treatment did not affect the morphology of the EVs - a concern raised by some researchers with regards to alternative active loading techniques such as electroporation.[54] Despite these positive findings, detergents have not been widely applied in EV loading studies. One reason for this may be that detergents such as saponin are difficult to remove from EV solutions, which poses potential problems with downstream measurement of loading efficiency and the identification of surface proteins. [65] While the inclusion of washing steps required for the removal of any residual detergent would necessitate additional time-consuming wash steps, potentially increasing the loss of EVs and further affecting reproducibility. Furthermore, despite observations that detergent permeabilisation does not negatively impact EV morphology; to the authors’ knowledge, no study has yet sought to investigate disturbances in the organisation of membrane microdomains such as lipid rafts or tetraspanin-enriched microdomains resulting from incubation with detergents. Consequently, the downstream effects of these processes on EV binding and uptake required for the transfer of cargo cannot be eliminated and further studies directly comparing the biodistribution and uptake of EVs is required.

Sonication applies ultrasound to induce mechanical disruption via the process of cavitation. The process may be coupled with repeated freeze/thaw cycles, in which the formation of ice crystals aid in the temporary disruption of the membrane and assisting the encapsulation of therapeutic cargoes.[66] Sonication was applied in approximately 14% of EV loading studies covered in this review, almost exclusively for the loading of hydrophobic chemotherapeutic agents such as paclitaxel and doxorubicin. For example, this method has been used to load macrophage-derived vesicles with both catalase[54] and paclitaxel.[67] In both studies a probe sonicator was applied to administer 6 cycles of sonication at 20% amplitude. Comparatively high loading efficiencies were achieved using sonication (26% loading of catalase and 28% loading of paclitaxel) in comparison to other loading methods and despite the different physical and chemical properties of the two molecular cargoes, remarkably similar loading efficiencies were achieved. Outcomes perhaps suggest that the non-specific and disruptive nature of the sonication method facilitates the indiscriminate loading of a wide range of diverse molecular cargos. However, given the size of the enzyme catalase (240 KD) it is clear that a large EV concentration would be required to achieve a modest loading efficiency. However, the precise number of EVs applied in the catalase study is not defined, which makes the efficiency of the study difficult to validate. In addition to using a tuneable probe, sonication can also be achieved using a standard laboratory ultrasonic bath, which makes the procedure simple and achievable even in the most basic laboratory setting. Lamichhane *et al* demonstrated loading of small interfering RNAs into EVs isolated from human embryonic cells by UC using this method, evidencing 325% (an increase of over 20 pmol per 100 µg EVs) greater loading when compared with unsonicated controls, but with no description of loading efficiency or biodistribution.[68] However, this process is limited since it is often not possible to control the settings of an ultrasonic bath, thereby restricting optimisation. Furthermore, it is unclear whether the effects of sonication are equally disruptive for EVs of varying sizes or whether the wave lengths applied using this method are only appropriate for the disruption of larger EVs, with further studies required.

Although it is evident that mild sonication (2–35 kHz) has demonstrated success in the active loading of EVs, optimum loading conditions (for example relating to amplitude, sonication time and number of cycles) have not been fully investigated. Furthermore, it has been suggested that certain cargo types (such as RNAs) may experience aggregation or degradation as a result of the loading procedure.[69] Lamichhane *et al* found that prolonged sonication of EVs for the loading of siRNA and plasmid DNA caused damage to the molecular cargo, with degradation of plasmid DNA, while sonication for shorter time periods induced minimal damage.[68] This will inevitably have an effect when determining loading efficiency as well as potentially impacting downstream therapeutic efficacy. It is also pertinent to highlight the fact that existing studies have not verified whether sonication induces changes in the composition and topography of the EV membrane. For instance, mechanically induced exposure of the lipid phosphatidylserine on the EV outer leaflet could result in an altered biodistribution through its recognised binding of opsonins that direct phagocytic uptake.[70] While milder sonication is less likely to negatively affect the integrity of EVs/cargo, this process is frequently coupled with freeze/thawing, which can negatively impact EV integrity.[71] Repeated freeze/thawing has been shown to cause degradation of RNA and proteins, which could in turn affect numerous aspects of EV mediated drug delivery such as targeting, binding/internalisation (i.e. the viability and properties of surface ligands) and the activity of any incorporated cargo.[72] While the effects of freeze/thaw cycles on EV proteins and RNA has not been thoroughly investigated, we would advocate that when applying this process for EV loading, - whether supplementing other loading strategies or used as a standalone technique - authors need to consider the potential effects on EV integrity and observed tendency for some EVs to aggregate when applying freeze/thawing, ideally providing data to evidence vesicle/cargo functionality.

Electroporation functions by applying a transient electric field to permeabilize the membrane and enable the passage of exogenous molecules, with loading efficiency being inversely proportional to the size of the molecules being loaded.[73] Following the removal of the electric field, membrane resealing occurs, encapsulating molecules within the vesicle. Electroporation has been widely applied as a means of loading cells with cargo since the 1980s, when Neumann *et al* used a custom-built electroporation chamber to transfect DNA into murine cells.[74] In recent years the technique has been extensively applied in EV studies, with the majority of publications utilising this method (approximately 75% of studies covered in this review, see Table 1 and S1). Although controversies abound regarding the application of electroporation in EV studies - and will be discussed shortly - many publications have applied this method for the loading of RNAs, utilising EVs isolated from a diverse range of sources including plasma,[5] macrophages,[67] breast cancer cells,[64] embryonic stem cells,[64] mesenchymal stem/stromal cells,[64] C2C12 cells,[16] lymphocytes,[75] HeLa cells,[7], [76], [77] endothelial cells,[64], [78] and red blood cells.[79] Much like passive and active approaches previously detailed in this review, electroporation has been widely applied for the encapsulation of chemotherapeutic drugs. The chemotherapy drugs doxorubicin and paclitaxel have been most frequently studied in this context, with examples of loading observed in EVs derived from HeLa cells,[7] dendritic cells,[9] macrophages,[67] and embryonic kidney cells.[8], [80] In these studies, a variety of loading efficiencies have been achieved, ranging from 0.5% up to 20%. However, this method has also been broadly applied to load a wide range of other candidate drugs, RNAs and DNA (Table 1 and S1), with reported loading efficiencies ranging from < 1% [7], [80], [81], [82], [83] to over 60%.[85] Notably, there are some instances in which researchers have even failed to load EVs using electroporation, while others have not provided relevant data for assessments to be made.[83], [84], [86] The efficiency of this method is likely to be heavily dependent upon a range of factors, relating to EV composition, the biochemical properties of cargo being loaded and the electroporation parameters employed (voltage, pulse length and pulse number). Cargo size is also an important factor in EV loading, with dsDNA across a range of 250–4000 bp in length being loaded, while minimal internalisation of 4,000 bp sized dsDNA was observed - although exact loading efficiencies were not reported in this study.[83] However, a current lack of standardised electroporation parameters and inconsistencies in the reporting of study methodologies/outcomes continue to make comparisons challenging. Moreover, much like other previously discussed loading techniques, it remains to be proven whether many of these approaches can achieve authentic luminal loading or whether the therapeutic content is merely associated with the EV. This important distinction can be made by incubating the ostensibly loaded EV fraction in the presence of RNase/proteinase or using density gradient centrifugation – in which the cargo should float with EVs into the same gradient.

Schwan’s equation implies that an inversely proportional relationship exists between the radius of a spherical cellular object and the minimum magnitude of the electric field required to achieve loading.[87] Although this dependence has not always been precisely observed due to a complex interplay between membrane conductivity, permeability and transmembrane voltage, it could provide an initial foundation for defining appropriate electrical fields for application in EV loading studies. It is possible that EVs isolated from different sources and derived via different pathways of biogenesis will exhibit different encapsulation and retention efficiencies – a recurring question raised in the aforementioned 2017 ISEV online survey that has yet to be investigated.[19] Some electroporator manufacturers provide databases detailing optimal loading parameters for different cell lines and species. However, these parameters only pertain to the direct loading of cells and are typically for the loading of DNA and RNA. Lastly, commercial electroporators may utilise either square-wave or exponential decay pulses, allowing the user to modify different parameters depending on the instrument. Although square wave and exponential decay parameters can theoretically be converted to one another, no studies have compared the effects of different pulse types on EV electroporation or the effects of different commercial electroporation systems, thus the results of different systems are not fully understood.

In studies applying electroporation the accuracy of loading efficiency estimations has also been called into question, specifically due to an observed tendency for both EVs and their cargos to aggregate upon the application of voltage. In a study by Kooijmans *et al*, it was demonstrated that the electroporation process can result in the aggregation of siRNA, rendering the measurement of labelled siRNA inaccurate. Based on these findings, the study identified the process was considerably less efficient than previously described, with the actual loading efficiency of siRNA into EVs calculated to be less than 0.05%.[84] This was further supported by Lamichhane *et al*, who demonstrated siRNA aggregation was much more pronounced following electroporation when compared with alternative loading methods such as sonication.[68] In addition to the observed effects of electroporation on cargo aggregation, Johnsen *et al* showed that this process can also induce aggregation of EVs themselves, evidencing increases in particle sizes of adipose-derived stem cell EVs following electroporation (from 75 to 100 to 500 nm).[88] In light of these observations,[89] some researchers are now taking steps to prevent the aggregation of cargo, such as through the addition of EDTA to electroporated samples.[83] However, concerns have also been raised regarding the effects of electroporation on the integrity of EVs. In a study by Pomatto *et al*, the effects of this process on plasma-derived EVs was examined, demonstrating that certain voltages caused damage to the EVs.[4] However in a similar study, Fuhrmann *et al* found that electroporation had no effect on EV morphology, suggesting that electroporation can be used without causing substantial vesicle damage.[64] Fuhrmann *et al* also discovered a correlation between loading efficiency and the zeta potential of vesicles.[64] Consequently, more studies are required to accurately assess the effects of potentially disruptive active loading strategies on EV integrity and bioactivity.

Due to the diverse range of factors influencing the efficiency of EV loading, it is important that relevant details pertaining to the interpretation and reproducibility of experiments are consistently reported. There is a need to better define the effects of active loading strategies such as electroporation, sonication and chemical manipulation on the integrity and activity of both EVs and cargoes. Firstly, authors should acknowledge that loading strategies employed are often not optimal and could result in physical damage to EVs. As such, the inclusion of visual data could be valuable in supporting loading studies (i.e. to evidence that aggregation of EVs or therapeutic cargo is not occurring). On a basic level, some of this data can be achieved by providing representative TEM images, However, future studies providing detailed analyses of lipid/protein topography could be conducted to verify whether any unintended conformational changes are occurring during the loading procedure. Although we do not yet understand the degree to which conformational changes will inadvertently influence EV loading and cargo transfer, it is likely that manipulations induced during loading could inadvertently affect downstream analyses and biological applications - potentially reducing loading capacity, limiting tissue penetration and masking binding sites required for cellular internalisation and the transfer of active cargoes. As the field advances and translational applications begin to emerge, it is important that we begin to document adverse events associated with loading so that protocols can be modified accordingly. This information will be valuable in helping us to define an acceptable level of damage in which there will undoubtedly be a trade-off between loading and an acceptable level of downstream activity. Secondly, when applying active loading strategies, authors should endeavour to comprehensively report experimental parameters. For instance, when using electroporation, the voltage, pulse width and the number of pulses applied should be clearly documented. This should also be accompanied by basic information concerning buffers and vials used in the study. For both active and passive loading strategies, the concentration of molecular cargo used should be detailed and the number of particles defined. Attempts to further define the concentration of molecular cargo per particle or per EV-like particle (e.g. per CD9 positive particle number, which could be determined using flow cytometry or ELISA) would provide additional valuable insights that could be applied as a benchmark to improve reproducibility. Finally, the loading solution (e.g. PBS, organic solvent) used in each study should also be clearly identified to ensure experiments can be reproduced and loading efficiency can be calculated. Throughout the literature these factors are frequently not reported in sufficient detail, despite playing an important role in the outcome of EV loading studies. Based on the critical observations detailed in this section, an overview of recommendations pertaining to the loading of EVs is summarised in Table 2.

## 4 Assessment of EV loading

Following loading, EVs are typically isolated and washed to remove any free uninternalized or unbound cargo. In most cases UC, ultrafiltration [76], [82], [90] or SEC [64], [67] are used to isolate the loaded EVs prior to further analysis or, in fewer cases, a precipitating agent such as ExoQuick solution.[91], [92] Amongst the most common techniques used to assess loading is fluorescence spectroscopy, which is achieved by either harnessing the natural fluorescent properties of loaded drugs or fluorescently tagging cargoes.[3], [7], [9], [58], [59], [77], [93] However, inaccuracies associated with the leakage of dyes and altered loading resulting from direct interactions with molecular cargos cannot be ruled out at this stage.[94], [95] Flow cytometry has also been utilised to assess loading efficiency,[96] though in some cases this necessitates modification of the loaded vesicles, such as incubation with latex beads prior to analysis; thereby reducing the specificity of the technique.[79] It should also be noted that flow cytometry analyses performed on EV samples contain their own challenges and reporting strategy.[97] Studies loading DNA and RNA into vesicles often apply a lysis step to extract the EV-associated material and quantify it using commercial DNA or RNA assays.[83], [91], [92] But these procedures can be time-consuming and often do not reliably differentiate between loaded and surface-bound material. Lysis is frequently achieved through the addition of a lysis reagent or a detergent such as Triton.[80], [85], [98], [99] When applying detergents, authors should demonstrate that any reagents utilised to induce EV lysis can be removed from the resulting sample and/or do not interfere with methods applied for the quantification of cargo. A failure to do so may result in the inaccurate calculation of loading efficiency. Finally, some groups have quantified EV loading using high performance liquid chromatography (HPLC) - particularly in the case of studies applying chemotherapeutic agents.[51], [67] This method can provide an accurate, reproducible and high-throughput method for the quantification of target analytes. However the application of HPLC is typically restricted to the measurement of small molecules, with methods for the analysis of larger biomolecules (e.g. RNA) often requiring time-consuming sample preparation prior to analysis.[100]

The success of an EV-loading experiment can be expressed in one of three ways; loading efficiency, loading capacity, or via a specific *in vitro* or *in vivo* biological outcome (e.g. the activation of a signalling pathway or upregulation in a downstream molecule). Loading efficiency relates to the percentage of total available drug that has been encapsulated, whereas loading capacity refers to the amount of drug loaded per mass of particles.[101] However in EV studies these important indicators of efficiency are frequently unreported. This is particularly common for studies in which EVs are subsequently applied to *in vitro* or *in vivo* systems to monitor biodistribution and elicit a predicted biological outcome. In these cases, the success of the loading experiment is demonstrated largely on the basis of the observed biological response, such as in the case of the work by Shtam *et al*, where it was demonstrated that loaded EVs could deliver siRNA to target cells *in vitro* with evidence of post-transcriptional gene silencing in the RAD51 target gene.[52], [76] While the outcomes of these studies are valuable and support the hypothesis that EVs can be applied to deliver of functional RNA cargoes, the absence of important fundamental data relating to loading parameters and quantitative values to validate EV loading make it impossible to truly assess their efficiency and make reproduction challenging. An important consideration when applying EVs or any nanoparticle system in this context is understanding the loading capacity per EV. Unlike synthetic drug delivery systems, the comparative heterogeneity of EVs makes this calculation more challenging. However, attempts have been made to provide a theoretical estimate of loading capacity through the application of the Tammes Problem, which seeks to determine the packing of circles on a spherical surface.[102] Additionally, the intraluminal volume of an EV can be estimated from its size, and applied to approximate maximum loading capacity – providing the size of the cargo is defined.[84], [103] Application of this model could enable us to formulate an equation that provides a theoretical understanding of the absolute capacity of EVs and thereby provide an accurate benchmark to advance the design of EV loading studies.

## 5 Future perspectives

The lack of standardisation in EV loading studies and the high degree of variability in protocols applied throughout the literature calls for greater collaboration within the EV community to develop a set of standard resources and guidelines. Attempts have been made to improve standardisation in EV research, with initiatives such as the EV-TRACK database providing a platform for researchers to share and compare experimental parameters, with the aim of improving transparency in the reporting of EV studies and ultimately enhance interpretation and reproducibility.[48] While these databases enable simple searching of EV studies based on parameters such as cell species, isolation method, and analytical technique, there is currently no functionality for searching and comparing EV loading protocols. The addition of such information into EV-TRACK or the creation of a standalone database that serves as an open online repository for published loading protocols would provide a valuable means of standardisation that would have a positive impact at both a basic and translational level.

A major challenge in the development of reproducible techniques in EV research is ensuring the production of consistent batches of EVs, in terms of both purity and physical characteristics (i.e. size, surface markers). However, current methods for the production and analysis of EVs introduce considerable variation and often do not result in a reproducible fraction. One of the key disadvantages limiting the reproducibility of studies is the lack of suitable reference materials for the appropriate calibration and normalisation of data. In EV loading, this introduces variability that can render the quantification of EVs and the measurement of EV loading inaccurate, affecting the reproducibility and possible application of research findings. To this end, recombinant extracellular vesicles (rEV) that share physical and biochemical features with native EVs could be utilised as an appropriate biological reference material for the calibration of EV analysis methods (e.g. NTA, flow cytometry) and serve to accurately control the concentration of starting material.[104] The inclusion of these now commercially available rEVs could serve to reduce a key variable implicated in practically all loading studies, as well as for the standardised assessment of loading efficiency. Through the improved calibration and control of techniques used in the analysis of EVs, greater accuracy in EV characterisation could be achieved that is essential for the development of reproducible EV loading methods.

The use of alternative analytical techniques may improve the accuracy of assessing EV loading. Techniques such as Raman spectroscopy, mass spectrometry, ImageStream flow cytometry and super-resolution microscopy are robust analytical methods that could be readily applied to EV studies for the purpose of assessing loading efficiency. Raman spectroscopy is a technique which detects molecule-specific shifts in the wavelength of inelastically scattered light. It has already been applied for single vesicle analysis [105] and as a tool to rapidly assess the purity of EVs [106]. As such, it could have value in the assessment of EV loading efficiency. Mass spectrometry is a gold standard technique in analyte identification and quantification. It is widely utilised for the accurate and reproducible quantification of analytes. Gas chromatography-mass spectrometry and liquid chromatography-mass spectrometry have already been utilised in EV research for the compositional analysis of these particles, most frequently in proteomic and lipidomic studies. These techniques could provide a robust and reliable tool for the targeted quantification of molecular cargoes loaded into EVs, provided appropriate sample preparation is performed to remove contaminants that could interfere with the analytical techniques, such as detergents and buffered solutions. However, for mass spectrometry to provide a useful indication of EV loading, it is essential that the EV samples are of high purity and have not been isolated using methods such as PEG or commercial kit-based precipitation. Super-resolution microscopy is a high-resolution technique that could be applied for the imaging of EVs and the assessment of heterogeneity in loading. This method is also valuable for assessing the intracellular trafficking of EV material.[107] Lastly, ImageStream flow cytometry has been effectively utilised for the analysis of single EVs and the identification of distinct EV subpopulations.[39], [108] As such, it could offer advantages for the detection and monitoring cargo loading and transfer, particularly in the case of biological drugs such as proteins.

Finally, we would like to draw the reader’s attention to the fact that many similar challenges have been – and continue to be - faced by those working in the field of synthetic nanoparticle (NP) drug delivery. This field typically employs lipid or polymer systems for the encapsulation of therapeutic cargos; with the aim of increasing drug targeting, half-life, and therapeutic payload. To date, outcomes have resulted in multiple FDA-approved formulations for the treatment of diseases such as cancers (e.g. Doxil®), chronic kidney disease (e.g. Renagel®) and macular degeneration (e.g. Visudyne®).[109] However, despite notable successes in the nanoparticle filed, there have also been numerous failures and instances where market approval has been withdrawn due to concerns raised during clinical trials.[110] What is clear is that in the in the formulation of NPs, one-size does not fit all and much like natural EV systems, the unique properties observed at the nanoscale are often highly dependent on NP composition, surface properties, morphology, and the route of administration applied. This diversity has made drawing up a regulatory framework for nanomedicines highly complex and, to date, no standard reporting guidelines or standards have been universally agreed upon.[111] Consequently, nanomedicines are often regulated in the same manner as small molecule drugs. However, much like EV-based drug delivery systems, the pharmacodynamics of NPs will not be comparable to small molecule drugs and cannot be effectively regulated using the same framework. Position statements and critical reviews have highlighted a requirement for quality control (QC) checks that assess aspects such as drug release rate and biodegradation. In response to a growing need for standardisation and regulation in this multidisciplinary and relatively complex area of drug delivery science, dedicated laboratories have been established to provide robust characterisation and identification of crucial parameters related to the effectiveness and safety of nanomedicines. These laboratories include the Nanotechnology Characterization Laboratory (NCL) and European Nanomedicine Characterization Laboratory (EU-NCL). The former was founded in collaboration with the National Institute of Standards and Technology and the FDA to support the preclinical characterisation of nanomedicines with the aim of enhancing product manufacture and clinical translation. Standardisation in the field has also been supported by the establishment of international projects such as REFINE, which was founded to address challenges facing the regulation of diverse interdisciplinary medical products and medical devices based on nanomedicines and nanomaterials (http://refine-nanomed.eu).[111] As an emerging community of EV researchers, we have an opportunity to benefit from lessons learned in the formulation of FDA-approved NP drug delivery systems such as Doxil® and the community’s collective efforts to promote standardisation and engage with relevant stakeholders and regulators.[112] In the example of Doxil®, only by accounting for the low solubility of doxorubicin and high dosage required (tens to hundreds of mM) to achieve therapeutic efficacy could the researchers devise an appropriate and economically viable loading strategy. Undoubtedly, as the field of EV drug delivery advances it will begin to face many of the same hurdles as the NP field, with combinations of drugs/biologics exhibiting independent mechanisms of action (MoA) eventually being encapsulated in a single EV. This will add further complexity relating to the selection of complimentary drugs/biologics, their relative dosages and resulting toxicity. Even so, many of the core principles devised by early pioneers of synthetic liposome drug delivery systems such as Barenholz (e.g. drug/lipid ratio as a measure of utility) can be applied by scientists working with natural EV systems and we should take time to reflect on fundamental principles established in this aligned field of drug delivery science.[113] By reflecting on these core principles, we can begin to improve QC and drive clinically and commercially valuable advancements in the emerging field of EV drug delivery.

## 6 Conclusions

This article calls for standardisation in the reporting of experimental parameters that could influence the exogenous loading of EVs and provides relevant guidelines (Table 2) that broadly cover the five principal stages employed in a standard EV loading protocol: 1) EV isolation, 2) EV characterisation, 3) EV loading, 4) assessment of loading and 5) the functional delivery of cargo. The guidelines are not prescriptive and are designed to provide a template to encourage transparency and reproducibility in this rapidly evolving field. They have been developed in response to an acknowledged need for standardisation within the community. While we recognise that uncontrollable variation will inevitably be introduced through the application of distinct and varied EV isolation procedures available, attempts should be made to standardise the reporting of EV starting material (e.g. through the wider adoption of MISEV criteria and standardised assessments of EV purity) and authors are strongly encouraged to disclose essential methodological parameters (e.g. sonication or electroporation parameters) and comparative outcomes (e.g. loading efficiency and capacity) to allow for the wider reproducibility of findings and the collective optimisation of loading strategies. We would encourage attempts to distinguish the authentic loading of cargos from mere association with the EV membrane and for all studies to provide a measure of loading efficiency or a similar quantifiable indicator of efficacy. Lastly, when proposing a downstream biological/therapeutic outcome, it is important that authors validate the specificity of these effects using relevant potency assays and controls (e.g. cargo only and/or unloaded EVs). It is anticipated that the guidelines published in this review will provide a valuable foundation to help support the future development of a wider community endorsed criteria on the topic.

## Declaration of Competing Interest

The authors declare that they have no known competing financial interests or personal relationships that could have appeared to influence the work reported in this paper.
