# Supplementary material for: A call for the standardised reporting of factors affecting the exogenous loading of extracellular vesicles with therapeutic cargos
Source: Adv Drug Deliv Rev. 2021 Jun;173:479–91. doi: 10.1016/j.addr.2021.04.012 (PMC8191593; doi:10.1016/j.addr.2021.04.012)
Supplement: Supplementary data 1 [file mmc1.docx]

Table S1: Detailed overview of studies employing different loading methods and protocols.

| **EV Source** | **Loading Cargo** | **Loading Method** | **Loading Protocol** | **Loading Efficiency** | **Reference** |
| --- | --- | --- | --- | --- | --- |
| Immature dendric cells (murine) | Doxorubicin | Electroporation | 350 V, 150 µF | Loading efficiency <20% | [9] |
| Bronchoalveolar lavage fluid (murine) | miRNA | Electroporation | 5x 500 V, 10 ms | Not reported | [114] |
| Es. coli msbB mutant | siRNA | Electroporation | 700 V, 50 µF | Loading efficiency 15% | [115] |
| Ovarian cancer cells (human) | Plasmids | Electroporation | 2x 1000 V, 10 ms | Loading efficiency 1.75% | [134] |
| Cervical cancer cells (human) | Doxorubicin | Electroporation | 100V, 5 ms (poring pulse), 5x 20V, 50 ms, (transfer pulse). | Loading efficiency 0.5% | [7] |
| Red blood cells (human) | Oligonucleotides | Electroporation | 50-250 V, 100 µF | Loading efficiency 24% | [79] |
| Embryonic kidney cells (human) | siRNA | Electroporation | 400 V, 125 µF | Cargo retention <0.5% | [84] |
| Embryonic kidney cells (human) | Oligonucleotide & 5-Fluorouracil | Electroporation | 2x 1000 V, 10 ms | Loading capacity 3.1% for 5-FU and 0.5% for miR-21i | [116] |
| Embryonic kidney cells (human) | dsDNA | Electroporation | 2x 400 V, 125 µF | Loading efficiency ~2% | [83] |
| Cervical cancer cells (human) | Dextran & Saporin | Electroporation | 2x 5 ms (poring pulse), 5x 20V, 50 ms (transfer pulse) | Loading efficiency 0.4-0.5% | [82] |
| Embryonic kidney cells (human) | Doxorubicin | Electroporation | 250V, 125µF | Not reported. Expressed as amount loaded/particle (i.e. loading capacity). | [8] |
| B cell lymphoma (murine) | miRNA | Electroporation | Various voltages (0.130-0.200 kV), 100 μF | Loading efficiency 55% | [91] |
| Lymphocytes (human) | miRNA | Electroporation | 4x 1600 mV, 10 ms pulse | Not reported | [75] |
| Epithelial cells (human) | siRNA | Electroporation | 1x 1300 V, 10 ms | Loading efficiency 15.6% | [117] |
| Plamsa (human) | miRNA | Electroporation | 10x 750V, 20ms | Loading efficiency 31.63% | [4] |
| Primary endothelial cells (murine) | siRNA | Electroporation | 400 mV, 200 μF | Loading efficiency 15-25% | [78] |
| Monocytic cells (human) | miRNA | Electroporation | 3x 0-2000 V | Not reported | [118] |
| Monocytic cells (human) | miRNA | Electroporation | 150 kV, 100 μF | Not reported | [92] |
| Primary dendritic cells (murine) | miRNA | Electroporation | 20x 0.7 kV, 350 ms | Loading efficiency 3% | [90] |
| Plasma (human) | siRNA | Electroporation | 75-300 V, 100 μF | Max loading efficiency 35% | [5] |
| Embryonic kidney cells (human) | siRNA | Electroporation | 0.150kV, 100 μF | Not reported | [96] |
| Embryonic kidney cells (human) | Doxorubicin | Electroporation | 35 V, 150 μF | Loading efficiency 10% | [80] |
| Preosteoblast cells (murine) | Let-7 inhibitor | Electroporation | 150 V, 100 μF | Not reported | [136] |
| Bone marrow mesenchymal stem cells | miRNA | Electroporation | 1x 990 V, 40 ms | Not reported | [119] |
| Monocytic cells (human) | siRNA | Electroporation | 200 V | Loading efficiency 2% | [3] |
| Triple negative breast cancer cells (murine) | Oligonucleotides | Electroporation | 250 V, 100 μF | Not reported | [120] |
| Cervical cancer cells (human) | Dextran & Saporin | Electroporation | 2x 200 V, 5 ms, (poring pulse), 5x 20V, 50 ms (transfer pulse) | Loading efficiency 0.3% for dextran and 0.2% for saporin | [81] |
| Milk (bovine) | Puromycin | Electroporation | 250 V, 950 μF | Not reported | [137] |
| Mesenchymal stem cell (murine) | miRNA & Mimics | Electroporation | 350 V, 150 μF | Not reported | [121] |
| Primary dendritic cells (murine) | siRNA | Electroporation | 400 V, 125 μF | Cargo retention <25% | [16] |
| Mesenchymal stem cell (murine) | Oligonucleotides | Electroporation | 0.2 kV, 100 μF | Loading efficiency max ~62%. | [85] |
| Monocytic cells (human) | miRNA | Electroporation | 150 kV, 100 μF | Not reported | [99] |
| Adipose tissue (human) | miR-10a Mimic | Electroporation | 2x 400 V, 100 µs | Not reported | [122] |
| Embryonic kidney cells (human) | CRISPR/Cas9 | Electroporation | 200 mV, 400 µF, 200Ω, 20 ms. | Not reported | [141] |
| Chondrocyte (human) | miRNA (miR-140 mimic) | Electroporation | 350 V, 150 µF | Loading efficiency 60% | [123] |
| Breast and colon cancer (human) | Doxorubicin | Electroporation | 150 V, 0.125 × 1000 μF | Loading efficiency 1.50% | [138] |
| Fibroblast (human) | siRNA | Electroporation | 400 V, 125 μF | Not reported | [124] |
| Umbilical mesenchymal stem cells (human) | miRNA mimic | Electroporation | Not specified | Not reported | [125] |
| Teratoma cells (murine) | Plasmid | Electroporation | 1000 V, 5 ms, x1 | Loading efficiency 20% | [135] |
| Bone marrow mesenchymal stem cells (human) | Antisense oligonucleotides | Electroporation | 3x 400 V and 25 μF | Loading efficiency 25.4% | [126] |
| Monocytic cells (human) | miRNA | Electroporation & Chemical Transfection | Electroporation: 5x 0.5 kV, 10 ms, x5  Calcium Chloride: Ice/heat shock with CaCl2 | Not reported | [127] |
| Milk (bovine) | siRNA | Electroporation & Chemical Transfection | Electroporation: 400 V, 125 μF, 10-15 ms.  Incubation: With transfection agent for 10 mins at 37^o^C. | Loading efficiency 30% for Exo-Fect, 4-5% for electroporation | [6] |
| Embryonic kidney cells (human) | siRNA/miRNA/nucleic acid | Electroporation & Sonication | Electroporation: 2x 400 V, 125 µF  Sonication: 30 sec at 35 kHz x2 | Not reported. Expressed as amount loaded/particle. | [68] |
| Umbilical cord blood macrophages (human) | Cisplatin | Sonication | 20% amplitude, 6 cycles of 30 s on/off for 3 min | Loading capacity 28-30% | [130] |
| Raw 264.7 macrophages (murine) | Doxorubicin | Sonication | 20% amplitude, 6x 30s on & 150s off | Loading efficiency 8-11% | [93] |
| Brain endothelial cells (murine) | Paclitaxel & Doxorubicin | Incubation | 2 hours at 37^o^C | Not reported. Expressed as amount loaded/particle. | [50] |
| Prostate cancer cells (human) | Paclitaxel | Incubation | 1 hour at 22^o^C | Loading efficiency 9.2% | [51] |
| Wharton’s-jelly derived mesenchymal stem cells | hsiRNA | Incubation | 1 hour at 37^o^C | Not reported. Expressed as amount loaded/particle. | [58] |
| Primary glioblastoma cells (human) | hsiRNA | Incubation | 90 mins at 37^o^C | Loading efficiency ~80% max (EV-associated, may include unloaded) | [59] |
| Wharton’s-jelly derived mesenchymal stem cells | hsiRNA | Incubation | 1 hour at 37^o^C | Loading efficiency ~70% max (EV-associated, may include unloaded) | [60] |
| Milk (bovine) | Paclitaxel | Incubation | 15 mins at room temperature | Loading efficiency 8% | [28] |
| Neuroblastoma cells (murine) | cc-siRNA | Incubation | 30-60 mins at 4-42^o^C | Loading efficiency max 75% | [128] |
| Milk (bovine) | Various chemopreventive agents & chemotherapeutic drugs | Incubation | Room temperature | Loading efficiency 10-40% | [133] |
| Mammary, prostate, colon, & liver cancer (human) | Saponins & flavonoids | Incubation & electroporation | Incubation (1 hour), Electroporation (200 V, 50 μF and 400 V, 50 μF) | Electroporation (up to 49.56%), incubation (up to 66.89%) | [129] |
| hMSCs, HUVECs, hESCs, MDAs (human) | Porphyrins | Incubation & Electroporation | Incubation for 10 mins at RT. Electroporation 200 Ω, 200 mV, 500 μF, 20–30 ms. Extrusion with lipid extruder. Saponin-assisted loading with 0.1mg/mL saponin. | Loading efficiency 0.6 to 35-fold increase | [64] |
| Raw 264.7 macrophages (murine) | Catalase | Incubation, sonication, freeze-thaw, extrusion | Incubation: Room temperature (with and without saponin)  Freeze/Thaw (3x)  Sonication: 6x 500 V, 2 kHz, 20% power  Extrusion: Lipid extruder | Loading efficiency 4.9% for incubation, 18.5% for saponin incubation, 26.1% for sonication, 22.2% for extrusion, 14.7% for freeze/thaw | [54] |
| Raw 264.7 macrophages (murine) | Paclitaxel & Doxorubicin | Incubation & sonication | Various protocols assessed | Not reported. Expressed as amount loaded/particle. | [131] |
| U937 (human) | Doxorubicin | Incubation, saponin-assisted incubation, freeze/thaw | Incubation at RT for 24 hours, incubation at 37˚C for 5 mins, incubation with 0.2% saponin for 5 mins, freeze/thaw x3. | 24 hour incubation ~13%, 5 min 37˚C incubation ~16.5%, saponin permeabilsation ~50%, freeze/thaw ~13%. | [61] |
| Peritoneal macrophage cells (murine) | Tripeptidyl peptidase-1 | Sonication, saponin-assisted incubation | Sonication (30 mins), incubation (30 min incubation with saponin) | Not reported | [139] |
| Melanoma cells (murine) | Hollow gold nanoparticles | Incubation, thermal shock, sonication, electroporation | Incubation at RT, Incubation with 0.2% saponin, thermal shock (freeze/thaw and heating), sonication (6 cycles at 2 kHz, 4 seconds on/2 seconds off). Electroporation (1x 950 µF) | Loading efficiency 7-19% | [63] |
| Raw 264.7 macrophages (murine) | Paclitaxel | Incubation, Sonication & Electroporation | Incubation: Room temperature  Electroporation: 1000 kV, 5 ms  Sonication 6x 30 sec on/off | Loading efficiency 1.4% for incubation, 5% for electroporation, 28% for sonication | [67] |
| Endometrial stem cells (human) | Atorvastatin | Freeze-thaw, sonication, incubation | Freeze-thaw (3x -80), sonication (500 V, 2 kHz, 20%), incubation (with & without tween-20) | Loading efficiency: freeze/thaw ~10%, sonication ~20%, incubation ~20%, incubation with tween 28% | [132] |
| Breast cancer & umbilical vein endothelial (human) | Nucleic acid nanoparticles | Chemical transfection | Not specified | Not reported | [140] |
| Malignant ascitic fluid (human) | siRNA | Chemical transfection & Electroporation | Chemical transfection: Incubation with reagent for 10 mins at room temperature  Electroporation 20x 0.7 kV, 350 ms | Not reported | [76] |
